# Insights into Therapeutic Response Prediction for Ustekinumab in Ulcerative Colitis Using an Ensemble Bioinformatics Approach

**DOI:** 10.3390/ijms25105532

**Published:** 2024-05-18

**Authors:** Kanellos Koustenis, Nikolas Dovrolis, Nikos Viazis, Alexandros Ioannou, Giorgos Bamias, George Karamanolis, Maria Gazouli

**Affiliations:** 1 Gastroenterology Department, Evangelismos-Polykliniki General Hospital, 115 27 Athens, Greece; k.koustenis@yahoo.gr (K.K.); nikos.viazis@gmail.com (N.V.); 2 Laboratory of Biology, Department of Basic Medical Sciences, Medical School, National and Kapodistrian University of Athens, Michalakopoulou 176, 115 27 Athens, Greece; ndovroli@med.uoa.gr; 3 Gastroenterology Unit, Alexandra Hospital, 115 28 Athens, Greece; 4 GI-Unit, 3rd Academic Department of Internal Medicine, National and Kapodistrian University of Athens, Sotiria Hospital, 115 27 Athens, Greece; gbamias@gmail.com; 5 Gastroenterology Unit, Second Department of Surgery, Aretaieio Hospital, Medical School, National and Kapodistrian University of Athens, 115 27 Athens, Greece; georgekaramanolis@yahoo.co.uk

**Keywords:** ulcerative colitis, ustekinumab, bioinformatics, prognostic markers

## Abstract

Introduction: Optimizing treatment with biological agents is an ideal goal for patients with ulcerative colitis (UC). Recent data suggest that mucosal inflammation patterns and serum cytokine profiles differ between patients who respond and those who do not. Ustekinumab, a monoclonal antibody targeting the p40 subunit of interleukin (IL)-12 and IL-23, has shown promise, but predicting treatment response remains a challenge. We aimed to identify prognostic markers of response to ustekinumab in patients with active UC, utilizing information from their mucosal transcriptome. Methods: We performed a prospective observational study of 36 UC patients initiating treatment with ustekinumab. Colonic mucosal biopsies were obtained before treatment initiation for a gene expression analysis using a microarray panel of 84 inflammatory genes. A differential gene expression analysis (DGEA), correlation analysis, and network centrality analysis on co-expression networks were performed to identify potential biomarkers. Additionally, machine learning (ML) models were employed to predict treatment response based on gene expression data. Results: Seven genes, including BCL6, CXCL5, and FASLG, were significantly upregulated, while IL23A and IL23R were downregulated in non-responders compared to responders. The co-expression analysis revealed distinct patterns between responders and non-responders, with key genes like BCL6 and CRP highlighted in responders and CCL11 and CCL22 in non-responders. The ML algorithms demonstrated a high predictive power, emphasizing the significance of the IL23R, IL23A, and BCL6 genes. Conclusions: Our study identifies potential biomarkers associated with ustekinumab response in UC patients, shedding light on its underlying mechanisms and variability in treatment outcomes. Integrating transcriptomic approaches, including gene expression analyses and ML, offers valuable insights for personalized treatment strategies and highlights avenues for further research to enhance therapeutic outcomes for patients with UC.

## 1. Introduction

Ulcerative colitis (UC) is a chronic inflammatory bowel disease (IBD), characterized by an intermittent and often recurrent course [1]. Its pathogenesis is a complex combination of genetic, immunological, and environmental factors [2]. The prevalence of UC is higher in Western societies, with newly industrialized countries experiencing a significant rise in its incidence [3]. Given the chronic nature of the disease, prolonged treatment is frequently necessary, accompanied by frequent hospitalizations that impact patients’ quality of life and contribute to the strain on healthcare systems [4,5].

Monoclonal antibodies targeting tumor necrosis factor-α (TNF-α) have revolutionized treatment, emerging as the most effective and well-established option for UC [6]. As our understanding of its underlying mechanisms deepens, the range of available treatments expands, incorporating other biologic and innovative target therapies, such as monoclonal antibodies against the α4β7 integrin receptor, those suppressing the interleukin-mediated immune response, and molecules interfering with Janus kinase (JAK) pathways [7]. The advent of biological agents has marked significant progress in inducing and maintaining remission, reducing hospitalizations, and mitigating the need for surgery in severe or recurrent cases. Nevertheless, more than half of patients exhibit inadequate responses or a loss of response to any available treatment over time [8,9]. Ustekinumab is a human IgG1 monoclonal antibody to the p40 subunit of IL-12 and IL-23 that has been recently approved for treatment of adults with UC. The UNIFI clinical trial and subsequent real-world studies have proven its effectiveness and safety for the induction and maintenance of UC remission. However, only 40% to 58% of patients are expected to achieve symptomatic remission within the first 6 months of treatment, as per observational studies, leading to the precise selection of patients with a higher likelihood of responding to this treatment [10].

At present, there is a lack of definitive prognostic markers of response to any biological agent, posing a challenge in terms of personalized treatment strategies. Previous studies have attempted to correlate genetic polymorphisms found in IBD with response to biological therapies, but these correlations have been weak, and the use of other methods of molecular biology and genetics, such as transcriptomics technologies, are promoted for further research [11,12]. Several attempts have been made to describe the transcriptional landscape in patients with UC, mainly by hybridization methods, such as microarrays of nucleotide oligomers [13,14]. Recent data indicate variations in mucosal inflammation patterns and serum cytokine profiles between responders and non-responders to biologic therapy. Studies have primarily focused on anti-TNF agents [15,16]. Similar studies involving vedolizumab show that its efficacy is linked to specific gene expression profiles prior to treatment and the dysregulation of specific pathways of inflammation [17,18].

Given the recent approval of ustekinumab for UC, a notable gap exists in transcriptional studies specific to this drug, with the current prognostic models including mainly clinical parameters with vague results [10]. The aim of our study was to investigate prognostic markers of response to ustekinumab of patients with UC, utilizing information from their colonic transcriptome before treatment initiation.

## 2. Results

### 2.1. A Seven-Gene Signature Has the Potential to Predict Response to Ustekinumab

**Figure 1:**
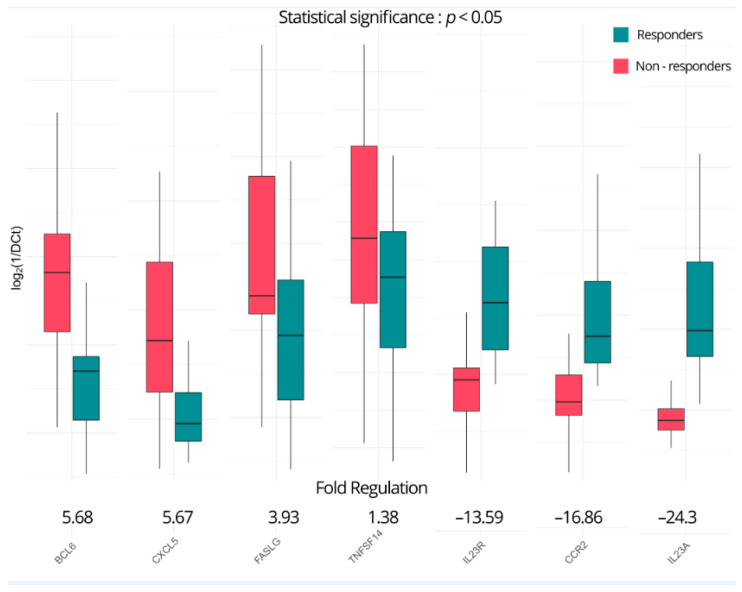
Genes significantly (*p* < 0.05) differentially expressed between ustekinumab responders and non-responders at baseline.

As described in our methodology, a DGEA was performed for gene expression at baseline for the two sample groupings of the responders, who were used as controls, and the non-responders. Only seven genes, as presented in Figure 1, effectively achieved *p* < 0.05 due to what appears to be a high level of variability. In the non-responders, B-cell lymphoma 6 (*BCL6*), C-X-C motif chemokine ligand 5 (*CXCL5*), and Fas ligand (*FASLG*) show positive fold upregulations of 5.68, 5.67, and 3.93, respectively. Tumor necrosis factor ligand superfamily member 14 (*TNFSF14*), while achieving statistical significance, displays a modest upregulation of just 1.38-fold. On the other hand, interleukin-23 receptor (*IL23R*), C-C chemokine receptor type 2 (*CCR2*), and interleukin-23 subunit alpha (*IL23A*) are significantly downregulated with fold regulations of −13.59, −16.86, and −24.3, respectively. While these seven genes provide strong indications of their ability to serve as predictive biomarkers, we wanted to expand our investigation to include all genes that present two or more times up- or downregulation to better understand the inflammatory and immunological backgrounds of these patients.

**Figure 2:**
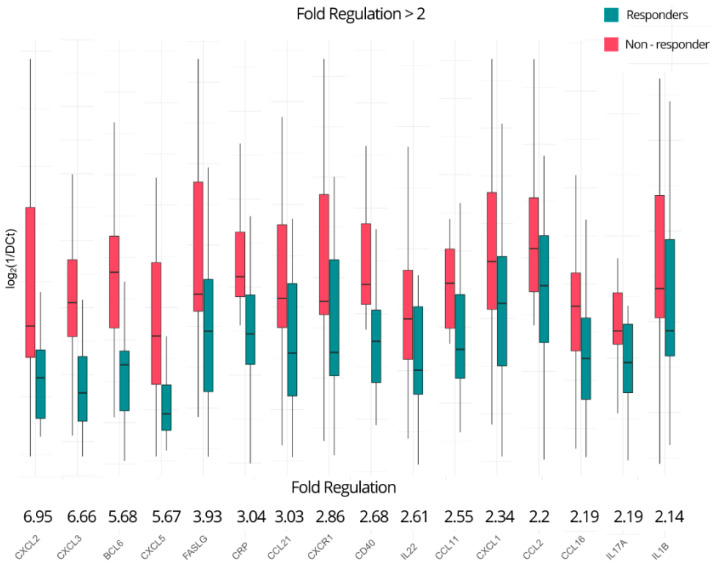
Genes upregulated in non-responders at baseline of ustekinumab with a fold regulation of at least 2.

**Figure 3:**
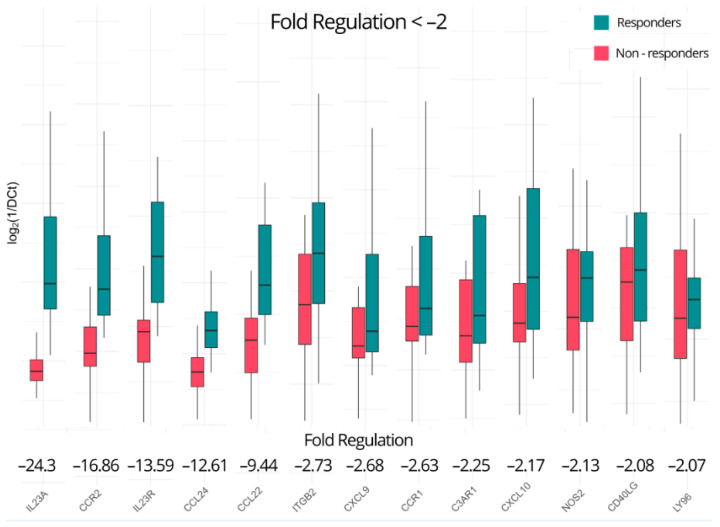
Genes downregulated in non-responders of ustekinumab at baseline with a fold regulation of at most −2.

In total, 29 out of the 84 investigated genes appear to be dysregulated using the aforementioned criteria, including six of the seven statistically significant genes. In detail, C-X-C motif chemokine ligand 2 (*CXCL2*) and C-X-C motif chemokine ligand 3 (*CXCL3*) show the highest positive fold regulations of 6.95- and 6.66-fold, respectively, indicating significant perturbation in the non-responders. *BCL6*, *CXCL5*, *FASLG*, C-reactive protein (*CRP*), C-C motif chemokine ligand 21 (*CCL21*), C-X-C motif chemokine receptor 1 (*CXCR1*), CD40 molecule (*CD40*), interleukin-22 (*IL22*), C-C motif chemokine ligand 11 (*CCL11*), C-X-C motif chemokine ligand 1 (*CXCL1*), C-C motif chemokine ligand 2 (*CCL2*), C-C motif chemokine ligand 16 (*CCL16*), interleukin-17A (*IL17A*), and interleukin-1 beta (*IL1B*) also show expression upregulations ranging from 2.14- to 5.68-fold (Figure 2). As for the downregulated genes (Figure 3), lymphocyte antigen 96 (*LY96*), CD40 ligand (*CD40LG*), nitric oxide synthase 2 (*NOS2*), C-X-C motif chemokine ligand 10 (*CXCL10*), complement C3a receptor 1 (*C3AR1*), C-C motif chemokine receptor 1 (*CCR1*), C-X-C motif chemokine ligand 9 (*CXCL9*), integrin subunit beta 2 (*ITGB2*), C-C motif chemokine ligand 22 (*CCL22*), C-C motif chemokine ligand 24 (*CCL24*), *IL23R*, *CCR2*, and *IL23A* are downregulated, ranging from −2.07-fold to an impressive −24.3-fold, as previously noted. A summary of all the up- and downregulated genes in the non-responders with a fold regulation of at least ±two-fold is presented in Table 1.

### 2.2. Inflammation- and Autoimmunity-Related Gene Co-Expression Highlights Unique Biological Backgrounds of Responders and Non-Responders

**Figure 4:**
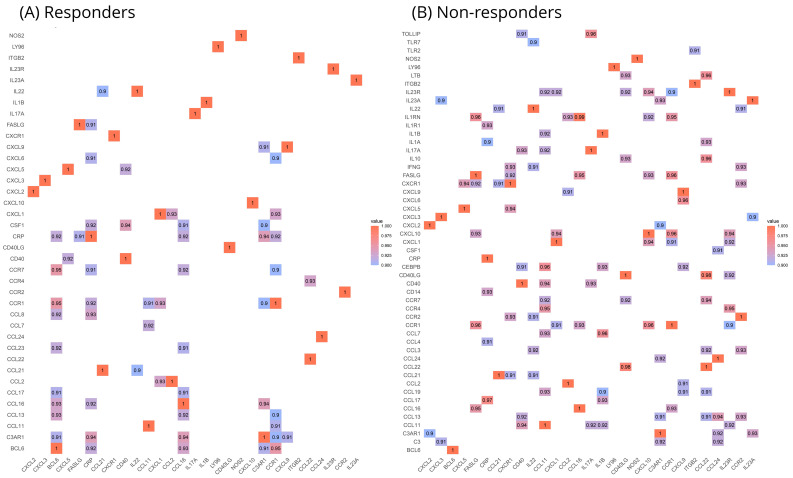
Correlation diagram of Spearman’s correlation coefficients (rho) for each pair of genes in our panel versus those found dysregulated with at least ±2-fold difference. (**A**) Correlations in responders to ustekinumab therapy at baseline (**B**) Correlation in non-responders to ustekinumab therapy at baseline. Values closer to 1 represent high correlations, and only those above 0.9 are shown.

In order to delve deeper into the potential biomarkers identified through the DGEA (those with a fold regulation of at least ±two and *TNFSF14*), within a broader biological framework, it is crucial to examine distinct correlation patterns and the networks derived from them, as elucidated by our analyses. We chose to examine each patient grouping separately, aiming to deduce the roles of specific genes in the context of each group. Figure 4 distinctly illustrates that the responders and non-responders manifest contrasting correlation patterns. In the former group, there appears to be less co-expression activity with only 38 genes presenting co-expression, while in the latter group, that number rises to 51. Regarding the initial seven genes highlighted from our DGEA, there is a significant difference between their correlation patterns; for example, *BCL6* is prominently featured in the responders’ results while its correlation activity is almost non-existent in the non-responders. The opposite can be observed for *IL23R* and *IL23A,* which are strongly correlated with many genes in the non-responders’ group and markedly sparse in the responders’ group. *FASLG*, *CXCL5,* and, to a lesser extent, *CCR2* appear to be equally implicated in the activity of both sample groups, while *TNFSF14* did not exhibit a strong correlation with any other gene in either group.

**Figure 5:**
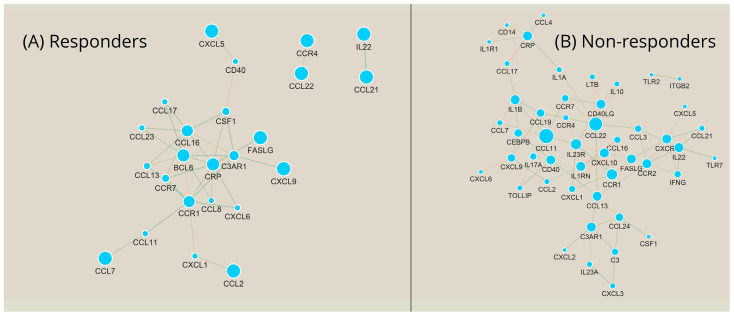
Co-expression networks based on the correlation values of Figure 4 in responders and non-responders. (**A**) Network of responders to ustekinumab therapy at baseline; (**B**) Network of non-responders to ustekinumab therapy at baseline. These networks were used for centrality analyses.

To help us explore all the correlations further and quantify the gene significance within each group, we constructed co-expression networks (Figure 5) and applied graph analysis metrics to them. In the responders’ group, *BCL6* and *CRP* are highlighted as the most important hubs with a degree centrality (DC) of 9, followed by *CCR1* and *CCL16* (DC = 8), *C3AR1* (DC = 6), and *CCR7* and *CSF1* (DC = 4). The results of betweenness centrality (BC) highlighted a number of genes as important mediators of the network. In ascending order, the top 10 are *CCR1*, *CRP*, *CSF1*, *BCL6*, *C3AR1*, *CCL16*, *CCL11*, *CD40*, *CXCL1*, *and CCR7* with respective BC values of 0.41, 0.27, 0.20, 0.17, 0.16, 0.12, 0.11, 0.11, 0.11, and 0.01. For the non-responders, the network, as expected from the corresponding correlation results, is much more complex. Interestingly, the top two hubs highlighted by this approach, *CCL11* (DC = 9) and *CCL22* (DC = 8), were not genes with a *p* < 0.05 dysregulation and showed only a moderate level of upregulation in the non-responders for the former and a moderate level of downregulation for the latter. The rest of the observed hubs include *IL23R* and *CCR1* with DC values = 6; *C3AR1*, *CRP*, *IL22*, *IL1B*, *CXCR1*, *CD40*, *CCR2*, *CCL13*, *CD40LG*, *FASLG*, *IL1RN*, and *CXCL10* with DC values = 5; and *CCL19*, *CXCL9*, *CCL24*, and *CEBPB* with DC values of 4. By examining the network bottlenecks based on their BC values, we observe the following genes in the top 10 spots: *CCL13* (BC = 0.35), *CCL22* (BC =0.33), *CCL11* (BC = 0.18), *CCR2* (BC =0.16), *IL23R* (BC = 0.15), *CD40* (BC = 0.14), *CRP* (BC = 0.14), *C3AR1* (BC = 0.13), *CXCR1* (BC = 0.13), and *IL1A* (BC = 0.11). Once more, we see *CCL11* and *CCL22* in these results, which highlights their crucial significance within the network. Studying the results of the two networks side by side, we also find some commonalities, like *CRP*, *CCR1*, and *C3AR1* as hubs in both and *CCR1*, *CRP*, *C3AR1*, *CCL11*, and *CD40* as shared bottlenecks.

### 2.3. Machine Learning Based on Expression Data Successfully Classifies Response to Ustekinumab

**Figure 6:**
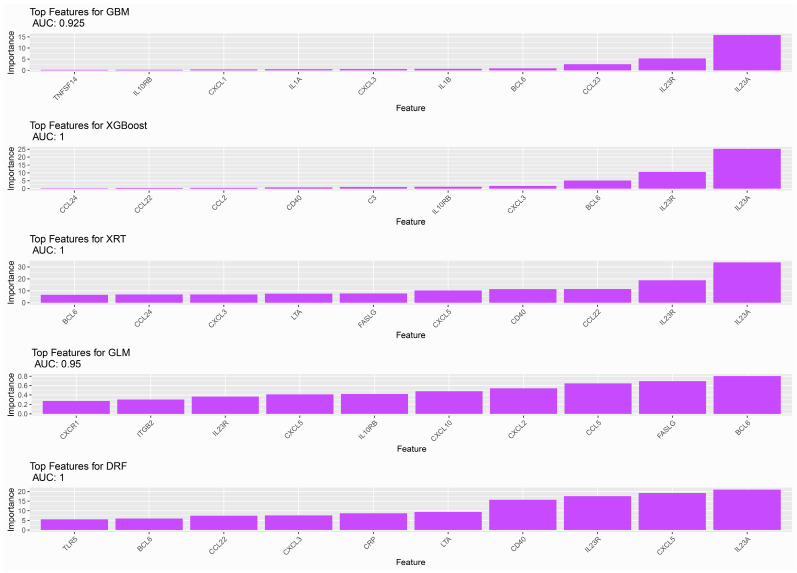
Machine learning results based on our expression data, showcasing the top features (genes) that can effectively predict response to therapy with ustekinumab using five different algorithms. For each of these algorithms, their area under the curve (AUC) values are shown.

As described previously, five different machine models were employed to test their classification power for our data. Their inherent ability to discern between different data groups, in our case, the expression data from the responders and non-responders to ustekinumab at baseline, and knowing the outcome a priori is the textbook definition of supervised learning. All the algorithms performed very well, as seen in Figure 6, albeit all suffering from the same restrictions of our relatively small dataset size. During all our tests, XGBoost performed consistently well with a minimal set of features and the ability to classify all of our samples by mainly taking into account the expressions of *IL23A*, *IL23R*, and *BCL6* and achieving an AUC of 1. It is worth noting here that all 84 gene expression values were provided to our models, highlighting the extreme edges of our DGEA spectrum that left *CCR2* out, for which the correlation analysis showed comparable levels of involvement between responders and non-responders and is a good indicator of its efficiency. XRT and DRF, both advanced implementations of random forests, exhibited variations in our tests but ultimately reached an AUC of 1 in the iteration depicted in Figure 6 with the specific set of features. It is worth noting here that DRF also included *LTA* and *TLR5* in its top features, two genes which were not found to be dysregulated by DGEA, while XRT only uses *LTA* in addition to other dysregulated genes. GBM and GLM achieve an AUC of 0.925 and 0.95, respectively, with GLM deviating in the importance of the features significantly from the other algorithms, reporting *CCL5*, *FASLG*, and *BCL6* as the top important features. Four of the algorithms, with the exception of GLM as mentioned previously, report *IL23A* as their most important feature, most probably because of its high dysregulation in the non-responders’ group.

## 3. Discussion

With the ongoing incorporation of newer therapeutic approaches like ustekinumab for the treatment of UC, the identification of prediction biomarkers for response is a key aim. In the present study, we focused on colonic mucosal tissues of UC in order to identify differential gene expression signatures that can predict responders and non-responders to ustekinumab therapy. Our multi-approach study of the gene expression of UC colonic mucosa reveals new insights into the pathophysiology mechanisms underlying the disease and might explain the variability in clinical outcomes.

Herein, we highlight a subset of seven genes with differential gene expressions that can effectively differentiate between non-responders and responders to treatment for samples obtained just before the initiation of ustekinumab. Non-responders were characterized by the upregulation of *BCL6*, *CXCL5*, and *FASLG*. It is known that *BCL6* mRNA is upregulated in UC patients compared to the healthy population, and it has been suggested that BCL6 can regulate the follicular helper T (Tfh) cells/follicular regulatory T (Tfr) cells ratio in the intestinal germinal center, promoting the development of UC, since an increase in the expression of *BCL6* mRNA suggests an increase in the number of Tfh cells [19]. Furthermore, ustekinumab therapy has been found to affect Tfh cell differentiation in Crohn’s disease in which Tfh cell frequencies decrease after the initiation of UST therapy in patients with clinical responses [20]. These studies help justify our findings of increased *BCL6* expression in non-responders. Similarly, CXCL5 is also reported to be upregulated in patients with IBD [21]. In further agreement with our findings, Pavlidis et al. [22] reported that the enrichment of IL-22-responsive transcriptional networks that include *CXCL5* is associated with poor response to ustekinumab therapy in patients with UC. Additionally, He et al. [23] suggested that *CXCL5* is among the significant DEGs that may be better predictors of ustekinumab non-response in patients with Crohn’s disease.

Regarding the association between *FASLG* mRNA expression and ustekinumab treatment failure, there are not yet any studies to clarify this. It is, however, known that FasL is expressed in CD3 lymphocytes infiltrating into UC, indicating that Fas-FasL-induced apoptosis contributes to the mucosal damage of ulcerative colitis [24]. It is possible that the dysregulation of apoptotic pathways, including those involving FASLG, may contribute to ustekinumab treatment resistance. Lastly, *TNFSF14* mRNA was found to be slightly upregulated in non-responders vs responders. Alternatively, *IL23A* mRNA expression was found to be significantly downregulated in non-responders at baseline. Our results are in agreement with the previous study by Nishioka et al. [25], which supported that diminished mucosal *IL23A* expression was mainly associated with ustekinumab resistance. Additionally, the fact that *IL23R* was also found to be significantly downregulated in non-responders can be explained by the preferential activation of memory T cells expressing *IL23R* by *IL23A* [26]. Regarding *CCR2*, it is known that it is a chemokine receptor involved in the migration of conventional dendritic cells (cDCs), mainly in the context of the colon [27], and that its interaction with C-C motif chemokine ligand 20 (CCL20), which is secreted by colonic cells, assists in the precise migration of cDCs to different parts of the GI tract, where they are involved in immune surveillance and maintaining immune balance [28].

Expanding our results, we have elucidated several more genes in patients with UC who are unresponsive to ustekinumab therapy. Among them, *CXCL2* and *CXCL3* showed significant levels of induction in non-responders, while lower levels of upregulation were observed for *IL22*, *IL17A*, *IL1B*, and *CXCL1*, which is in agreement with previous studies that show that the enrichment of IL-22-responsive transcriptional networks is associated with resistance to ustekinumab treatment in patients with UC [22].

To further our understanding of the immunological background of the resistance to ustekinumab therapy and unmask biological processes involved in these mechanisms, we performed a co-expression network analysis similar to what has recently been used to identify functional gene modules [29]. In responders, our analysis highlights *BCL6* and *CRP* as crucial hub genes, with *BCL6* implicated in regulating the differentiation and function of various immune cells, including B cells and T cells, and modulating inflammation-related signaling pathways [19,30]. Additionally, CRP is a known inflammatory marker [31] whose levels have been contradictorily associated with response to biological therapies. A negative correlation between CRP levels and response to anti-TNF therapies has been reported in UC [32]; however, these observations were not consistent with ustekinumab therapy [33]. Regarding non-responders, the crucial hubs involved the *CCL11* and *CCL22* genes. It is known that CCL11 is implicated in Th2 inflammatory diseases, and it has recently been reported that *CCL11* gene expression can be a predictive marker for ustekinumab response in patients with Crohn’s disease [23]. *CCL22* is constitutively expressed under homeostatic situations and inducible upon inflammation, while numerous immune cell types, like macrophages, dendritic cells (DCs), B cells, and T cells, secrete CCL22 upon activation [34]. While CCL11 is shared as a hub for both networks, indicating a more disease-centric role, CCL22 appears to be specific to non-responders. Regardless, the high involvement of both *CCL11* and *CCL22* as hubs and bottlenecks in our networks suggests their critical involvement in patients’ unresponsiveness to therapy and merits further investigation. The same can be said for other genes that are highly involved in regulating these networks while not significantly differentially expressed, like *CCL13*, which has previously been associated with response to α4β7 integrin antagonists [35], and *CCL16,* which is yet to be implicated in UC treatment but is involved in its pathophysiology [36].

The ML results of this study further reinforce the value of expression data in predicting response to ustekinumab. Almost all algorithms performed well, reaching AUC values of or close to one, but the XGBOOST and GBM algorithms overperformed by requiring only a short subset of genes to produce good results. This comes as no surprise since they are modern, robust algorithms that have shown good predictive results in previous works [37,38,39,40] using a variety of data as their input. These two algorithms are in agreement on the highly predictive nature of *IL23R*, *IL23A*, and *BCL6*, while GBM also utilizes *CCL23*, another staple of IBD pathophysiology [21], for its predictions. However, it has to be noted that even though ML approaches are in agreement with all the other approaches showcased in this study, they do suffer from the limited sample size, since they were created to process larger amounts of data.

While the preliminary findings of this study are promising, it is crucial to acknowledge the need for further research and independent validation to solidify our conclusions. This study, conducted on a limited number of patients within our available resources, offers valuable insights but may not capture the full spectrum of variability present in the broader population affected by UC. Expanding the scope of this research through larger sample sizes and diverse patient cohorts is essential to ensure the generalizability and reliability of our findings. By including a more extensive range of demographic, clinical, and genetic factors, future studies can better elucidate the factors influencing therapeutic response to biologics across different patient subgroups. Independent validation by other research groups is equally critical. Replicating the results in diverse clinical settings and populations can help confirm the robustness and reproducibility of the observed associations, reducing the risk of bias and increasing our confidence in the conclusions drawn.

Future research efforts should aim to address these limitations by employing rigorous study designs, incorporating long-term follow-up data, and employing advanced statistical methodologies to account for confounding variables.

Overall, despite these limitations, through a variety of computational approaches, our study showcases the predictive power of mucosal expression data in predicting patient response to ustekinumab treatment. We also highlight key genes that are supplementary to the therapeutic regimen to improve response that might be targeted in the future or that could be studied on more easily accessible samples (e.g., blood draws) to enable faster implementation.

Expanding on the critical need for biologics in treating ulcerative colitis (UC), it is essential to understand the profound impact these therapeutic interventions have on patients’ quality of life. When conventional treatments fail to elicit a response, patients often find themselves in a challenging cycle of switching from one therapy to another, which not only disrupts their daily lives but also adds emotional and financial burdens. In addition, biologics offer a targeted and often more effective treatment option by stabilizing disease activity and inducing remission, providing patients with the opportunity to regain control over their lives. This restoration of quality of life cannot be overstated, as UC can profoundly impact one’s daily activities, work productivity, and social interactions. The ability to predict therapeutic responses to these biologics holds immense promise in further optimizing treatment outcomes. The clinical applications of this work are manifold. Firstly, these markers will enable clinicians to personalize treatment strategies, tailoring interventions to individual patients based on their predicted response profiles. This not only enhances treatment efficacy but also minimizes unnecessary exposure to medications that may not yield the desired results. Furthermore, predictive response to biologics empowers patients by providing them with realistic expectations regarding treatment outcomes. Armed with this knowledge, patients can make informed decisions about their healthcare journey, potentially reducing anxiety and uncertainty surrounding their condition. From a healthcare system perspective, predicting therapeutic response to biologics can optimize resource allocation and healthcare spending. By identifying the patients who are likely to benefit most from specific biologic therapies, healthcare providers can streamline treatment pathways, reduce trial-and-error approaches, and ultimately improve patient outcomes while minimizing healthcare costs. In research settings, such predictive biomarkers offer invaluable insights into the underlying mechanisms of UC and may pave the way for the development of novel treatment strategies. By elucidating the factors that influence treatment response, researchers can uncover new therapeutic targets and refine existing treatment algorithms, ultimately advancing the field of gastroenterology and improving patient care.

## 4. Material and Methods

### 4.1. Patients

We performed a prospective, observational study of adult patients with a regular follow-up at Evangelismos, Sotiria, and Alexandra General Hospitals, three tertiary gastrointestinal (GI) centers in Greece. All patients were diagnosed with active UC and commenced treatment with ustekinumab between March 2022 and August 2023. The diagnosis of the disease was established based on well-defined clinical, endoscopic, and histological criteria [19]. The study was approved by the hospitals’ institutional review boards, and all patients provided their informed consent to participate (479/15-12-2022). They received an initial weight-based intravenous induction dose of ustekinumab (approximately 6mg/kg), followed by 90 mg of subcutaneous injection of the drug every 8 weeks, according to the standard administration protocol. Patients were evaluated at baseline, while predefined clinical and laboratory evaluations were recorded 6 months after treatment initiation. Before the start of ustekinumab therapy, biopsies were obtained during patients endoscopies from the most inflamed areas of the colon (usually at the site 20−30 cm proximal from the anal verge). The samples were obtained from each patient and placed immediately in RNALater buffer (Ambion, Austin, TX, USA) and preserved at −80 °C until further analysis. Disease activity was assessed by the Mayo score [41]. At 6 months post-treatment initiation, patients underwent repeated endoscopy to determine the endoscopic severity, and the total Mayo score was reevaluated. The main endpoint of our study was clinical remission at that timepoint, defined as a total Mayo score ≤ 2 with no subscore > 1.

The characteristics of responders and non-responders are shown in Table 2.

### 4.2. Differential Gene Expression Analysis (DGEA)

Total RNA was extracted from preserved mucosal biopsies using the Qiagen AllPrep RNA/DNA Mini Kit (Qiagen, Hilden, Germany). Subsequently, cDNA synthesis was carried out with the RT2 First Strand Kit (Qiagen) per the manufacturer’s instructions. Gene expression analysis was conducted using the Human Inflammatory Response & Autoimmunity PCR Array (PAHS-077Z, Qiagen) RT^2^ profiler with RT^2^ qPCR SYBR Green Master Mix (Qiagen) testing for a total of 84 genes.

Samples were divided into responders to therapy, used as our control group, and non-responders. Differential gene expression analysis (DGEA) was performed using the RT^2^ Profiler PCR Array Data Analysis software (version 3.5) from Qiagen. All samples met the quality criteria for PCR array reproducibility, RT efficiency, and absence of genomic DNA contamination. In brief, for within-sample normalization, the 2^−ΔCt^ method was applied using five housekeeping genes (*ACTB*, *B2M*, *GAPDH*, *HPRT1*, and *RPLP0*). Fold change (FC) was determined by the 2^−ΔΔCt^ method and is reported as the fold regulation in the results, which is more biologically relevant (genes exhibiting under-expression are denoted as the negative reciprocal of the fold change, while overexpressed genes are presented as the fold change). The statistical significance was assessed through Student’s *t*-tests conducted on the replicate 2^−ΔCt^ values for each gene in both responders’ and non-responders’ groups. All results were investigated based either on their statistical significance (*p* < 0.05) or on their fold regulation thresholds of <−2 or >2 (non-responders versus responders). DGEA plots were created in R v. 4.3.0 [42] for which log_2_(1/ΔCt) values were employed to normalize and highlight expression differences using the ggplot2 [43] package.

### 4.3. Gene Expression Correlation Patterns

Correlation analyses are crucial for understanding complex biological systems and highlighting potential relationships between genes. By examining co-expression patterns, we gain insights into the regulatory mechanisms underlying various biological processes and bring forth genes that exhibit coordinated expression changes across different conditions. To supplement our DGEA analysis and identify how the dysregulated genes found may interact with the rest of the genes in our panel, we employed an R-based pipeline. Our pipeline calculated the Spearman’s correlation coefficient (rho) for each gene pair and constructed a correlation matrix using the cor() function for each patient group (responders and non-responders). Spearman’s non-parametric test was selected, among the available correlation algorithms, based on the Shapiro–Wilk normality test, which showed that not all gene expressions were normally distributed. The correlation matrix was then filtered for only gene pairs exhibiting rho values less than −0.9 and higher than 0.9 to only capture strong relationships. Visualization of these correlations was produced using the ggcorrplot v. 0.1.4.1 package [44].

### 4.4. Analysis of the Co-Expression Network

Graph representation of genes correlated via their gene-expression produces a co-expression network capable of offering a holistic view of cellular processes by identifying key regulators and pathways. It enables the use of statistical network metrics, which are pivotal for uncovering the underlying structure and dynamics of gene interactions within biological systems. We used the Cytoscape v.3.10.1 [45] software to visualize and perform network analysis on the interactions of genes, based on our previous correlation analysis, separately in our sample groupings. Each gene represents a node on our network while its correlation coefficient is provided as the weight of the edges between each gene pair. We report biologically-relevant metrics [46,47], such as degree centrality (DC), which indicates the number of regulatory interactions among genes (high DC nodes/genes, called hubs, may be crucial regulators of gene expression and may have significant impact on cellular functions and phenotypes), and betweenness centrality (BC), which highlights nodes/genes that often acts as mediators of gene–gene interactions quantifying the extent to which a node/gene lies on the shortest paths between others in the network (often referred to as bottlenecks that control the flow of information). By quantifying the importance of these nodes/genes in responders and non-responders separately, we are able to discern specific patterns and genes that uniquely characterize each group and can potentially hold prognostic value.

### 4.5. Machine Learning Approaches

Finally, since our aim is to identify predictive markers to response to treatment, we employed an ML pipeline that enhances the DGEA. ML of gene expression data offers the advantage of uncovering complex patterns and relationships that may not be captured by traditional DGEA methods, allowing for a more nuanced and comprehensive understanding of biological processes while allowing us to see if specific markers can predict response to treatment and performance [48,49]. Our methodology is based on the h2o v.3.42.0.2 R package [50] and employs five different ML models, for which we calculate their area under the curve (AUC) metrics to measure their predictive power in discerning responders and non-responders to ustekinumab therapy. We report the AUCs and top 10 important features (genes) for each of the following algorithms: gradient boosting machine (GBM), XGBoost (eXtreme gradient boosting), extreme random trees (XRT), generalized linear model (GLM), and distributed random forest (DRF). The algorithms were selected for their robustness and previous effectiveness for biological data [51,52]. All models were trained using a five-fold cross-validation approach on 60% of our available samples (n = 23) and tested on the remaining 40% (n = 13) using datasets containing samples from both responders and non-responders.

## Figures and Tables

**Table 1:** Summary of all up- and downregulated genes in non-responders with a fold regulation of at least ±2-fold.

Gene	Fold Upregulation	Gene	Fold Downregulation
*CXCL2*	6.95	*IL23A*	−24.3
*CXCL3*	6.66	*CCR2*	−16.86
*BCL6*	5.68	*IL23R*	−13.59
*CXCL5*	5.67	*CCL24*	−12.61
*FASLG*	3.93	*CCL22*	−9.44
*CRP*	3.04	*ITGB2*	−2.73
*CCL21*	3.03	*CXCL9*	−2.68
*CXCR1*	2.86	*CCR1*	−2.63
*CD40*	2.68	*C3AR1*	−2.25
*IL22*	2.61	*CXCL10*	−2.17
*CCL11*	2.55	*NOS2*	−2.13
*CXCL1*	2.34	*CD40LG*	−2.08
*CCL2*	2.2	*LY96*	−2.07
*CCL16*	2.19		
*IL17A*	2.19		
*IL1B*	2.14		

**Table 2:** Clinoepidemiological characteristics of disease in patients with active UC at baseline who responded (n = 22) or did not respond (n = 14) to ustekinumab.

	Responders (n = 22)	Non-Responders (n = 14)	*p* *
Male (%)	16 (72.73)	10 (71.43)	0.932
Age, years, mean (SD)	48.43 ± 15.37	55.86 ± 19.37	0.205
Montreal classification, n (%)			0.721
*E1*	1 (4.55)	0 (0)	
*E2*	9 (40.91)	6 (42.86)	
*E3*	12 (54.55)	8 (57.14)	
Mayo score, median	7.5	7	0.517
Smoking status, n (%)			0.377
Never	8 (36.36)	3 (21.43)	
Former	4 (18.18)	1 (7.14)	
Active	10 (45.46)	10 (71.43)	
Anti-TNF exposed, n (%)			0.755
Yes	9 (40.91)	5 (35.71)	
No	13 (59.09)	9 (64.29)	
WBC, mean (SD)	8389.58 ± 3082.1	7951.67 ± 1917.6	0.656
CRP (mg/dL), mean (SD)	0.97 ± 1.11	1.95 ± 2.94	0.166
Platelets, mean (SD)	352,680 ± 118,288	274,727.3 ± 103,577	0.07
Hemoglobulin, mean (SD)	12.31 ± 1.71	13.45 ± 2.10	0.09

* indicates χ^2^ test, Abbreviations: WBC, white blood cells. Normal values are as follows: WBC 4000–11,000/μL; platelets 150,000–400,000/μL; hemoglobulin 13–17 g/dL for men and 12–16g/dL for women; albumin 3.5–5.5 g/dL.

## Data Availability

Data is contained within the article.
